# Inhibition of YB-1 phosphorylation enhances cisplatin activity and disrupts cell division in pleural mesothelioma

**DOI:** 10.1038/s41416-025-03177-0

**Published:** 2025-09-04

**Authors:** Karin Schelch, Nadine Maach, Muhammad Hashim, Benjamin Zitta, Dominik Kirchhofer, Gerald Timelthaler, Anna Solta, Dominik Emminger, Verena Kopatz, Mir A. Hoda, Walter Berger, Clemens Aigner, Balazs Dome, Glen Reid, Michael Grusch

**Affiliations:** 1 Center for Cancer Research, Medical University of Vienna, Vienna, Austria; 2 Department of Thoracic Surgery, Medical University of Vienna, Vienna, Austria; 3 Department of Radiation Oncology, Applied and Translational Radiobiology, Medical University of Vienna, Vienna, Austria; 4 National Koranyi Institute of Pulmonology, Budapest, Hungary; 5 Department of Thoracic Surgery, Semmelweis University and National Institute of Oncology, Budapest, Hungary; 6 Department of Translational Medicine, Lund University, Lund, Sweden; 7 Department of Pathology, Dunedin School of Medicine, Dunedin, New Zealand; 8 The Maurice Wilkins Centre, University of Otago, Dunedin, New Zealand

**Keywords:** Preclinical research, Mesothelioma

## Abstract

**Background:**

The cold-shock domain protein YB-1 is overexpressed in pleural mesothelioma (PM) and was shown to contribute to increased cell migration and platinum resistance.

**Methods:**

Phosphorylation of YB-1 at position serine 102 was analysed by immunohistochemistry, immunofluorescence and immunoblotting in PM tissue specimens and cell lines. Intracellular localisation experiments involved immunoblotting, transfection of fluorescent protein-tagged YB-1 and confocal imaging. YB-1 phosphorylation was inhibited with the RSK inhibitors BI-D1870 and LJH685. Effects of inhibition alone and in combination with radiation or cisplatin treatment were analysed by cell viability assays, clonogenic assays and videomicroscopy-based migration and cell fate map analyses.

**Results:**

YB-1 phosphorylated at serine 102 is present in PM cell lines and tissue. Inhibition of phosphorylation with BI-D1870 reduced YB-1 localisation in the cell nucleus and led to reduced cell viability, clonogenicity, migration and disrupted cell division. Moreover, exposure to BI-D1870 increased the effect of radiation and cisplatin treatment with additive to synergistic effects in PM cell lines and primary cultures.

**Conclusions:**

The serine 102 phosphorylated form of YB-1 contributes to the malignant phenotype of PM. Inhibition of YB-1 phosphorylation warrants further exploration as part of treatment strategies for this devastating disease.

## Background

Pleural mesothelioma (PM) is a very aggressive malignancy that arises from the mesothelial cells lining the pleural cavity [1]. It is often associated with asbestos exposure and characterised by rapid growth and therapy resistance. A combination of cisplatin and pemetrexed has been the standard of care chemotherapy for almost two decades [2]. More recently, dual immunotherapy consisting of a combination of nivolumab and ipilimumab has become a new first line therapy option [3, 4] but, like chemotherapy, often lacks efficacy [5]. Hence, the overall prognosis for PM patients has remained poor [3, 4] and additional treatment options are urgently required.

Y-box binding protein 1 (YB-1) is a multifunctional nucleic acid-binding protein that is involved in cellular stress response and drug resistance [6]. We have previously shown that YB-1 is a target of the microRNA miR-137 and its upregulation in PM correlates with loss of miR-137 [7]. Moreover, RNA-interference mediated knockdown of YB-1 as well as inhibition of YB-1 deacetylation by the histone deacetylase (HDAC) inhibitor entinostat reduced PM cell growth and survival, migration and chemoresistance [8, 9] demonstrating that YB-1 is a critical survival factor for PM cells.

YB-1 is phosphorylated at position serine 102 (S102) by ribosomal S6 kinase (RSK) and this phosphorylation has been shown to influence many of its biological functions although the results of different studies are conflicting. On the one hand, S102 phosphorylation of YB-1 was demonstrated to be essential for YB-1 nuclear localisation and therefore the associated oncogenic properties that arise from its transcriptional activity [10–12]. In line with this notion, the presence of high levels of S102 phosphorylated YB-1 correlated with poorer overall survival in patients with diffuse large B-cell lymphoma (DLBC) [13]. In melanoma on the other hand, it was found that the unphosphorylated cytoplasmic form of YB-1 is the main driver of its tumorigenic and prometastatic activity [14].

In PM, YB-1 S102 phosphorylation has not been investigated so far, despite the prominent role of YB-1 in this disease. Here we show that YB-1 is present in its S102 phosphorylated form (pYB-1) in cell models and tissues specimens of PM. Treatment with the RSK inhibitor BI-D1870 decreased S102 phosphorylation of YB-1 and reduced its presence in the cell nucleus. This resulted in increased cell size, cytokinesis failure and cell death as well as in decreased proliferation and migration. Moreover, BI-D1870 enhanced the efficacy of treatment with radiation and cisplatin, two frequently used treatment modalities in PM. These data suggest that pYB-1 represents a potential new target for improving PM management.

## Methods

### Tissue and cell models

Tissue specimens from PM and normal pleura were collected at the Medical University of Vienna. Tissue collection and analysis and establishment of cell cultures has been approved by the Ethics Committee of the Medical University of Vienna (EK #904/2009) and all patients have provided informed consent.

All PM cell models were either established at the Medical University of Vienna [15], purchased from ATCC, or provided by collaboration partners. Cells were cultivated in RPMI medium supplemented with 10% heat-inactivated fetal bovine serum (FBS) in a humidified atmosphere (37 °C, 5% CO_2_), authenticated by STR profiling [15, 16] and regularly checked for *Mycoplasma* contamination. Primary cultures were generated from surgically resected PM tumour tissue or pleural effusions at the Medical University of Vienna and used at <10 passages.

### Immunohistochemistry

Immunohistochemistry on formalin-fixed, paraffin-embedded patient-derived tumour and normal pleura tissue specimens was performed as previously described [17]. Blocks were cut into 4 µm slices and after deparaffinization and rehydration, antigen retrieval was performed in 10 mM citrate buffer (pH 6.0) for 10 min.

Samples were incubated with the following primary antibodies overnight at 4 °C: total YB-1 [18], (1:100), and pYB-1 S102 (#2900, Cell Signaling Technology (CST), Danvers, MA, USA; 1:200). Antibody binding was detected using the ImmPRESS® HRP Anti-Mouse IgG (Peroxidase) Polymer Detection Kit (Vector, Stuttgart, Germany) and Liquid DAB+Substrate (Agilent Dako) or the UltraVision LP detection system (Lab Vision Corporation, Fremont, CA, USA).

### Immunocytochemistry

PM cells (1.5 × 10^4^) were seeded in 8-well chamber slides (Thermo Scientific) and on the next day fixed with 4% PFA (Fluka Chemika) and stained as described [16]. Specifically, cells were incubated with pYB-1 S102 (#2900, CST; 1:100) overnight at 4 °C. Phalloidin-TRITC (P1951, Sigma-Aldrich, 50 µg/ml) and DAPI (D9542, Sigma-Aldrich, 1.5 µg/ml) were added together with the secondary antibody (Goat anti-Rabbit IgG 488, #35553, Thermo Fisher; 1:500). Slides were sealed using Vectashield mounting medium (Vector). Images were taken on an Olympus IXplore SpinSR Spinning Disk Confocal Microscope (Olympus).

### Protein isolation and Western blot

PM cells (2 × 10^5^ per 6-well or 1 × 10^6^ per 6 cm dish) were seeded and on the next day treated with BI-D1870, LJH685 or cisplatin as indicated. For total protein analysis, cells were harvested in lysis buffer (150 mM NaCl, 50 mM HEPES, 10% glycerol, 1 mM EDTA, 0.5 mM Na_3_VO_4_, 10 mM NaF, 1% Triton X100, 1.5 mM MgCl_2_). Nuclear and cytoplasmic fractions were isolated using the Ne-PER Nuclear and Cytoplasmic Extraction Reagents (Thermo Fisher, Waltham, MA, USA), as per the manufacturer’s instructions. Protein concentrations were determined using the BCA Protein assay (Bio-Rad, Hercules, CA, USA). Proteins were separated by SDS/PAGE and blotted onto PVDF membranes. Immunodetection was performed with the Clarity Western ECL Substrate kit (Bio-Rad) using antibodies against YB-1 (ab12148, Abcam, 1:1000) and pYB-1 (S102, C34A2, CST, 1:1000). Beta-actin (A5441, Sigma, 1:2000) or GAPDH (D18H11, CST, 1:1000) were used as loading controls for total and cytoplasmic protein and lamin B (AB-1, #NA12, Sigma, 1:1000) for nuclear fractions. HRP-coupled secondary antibodies were purchased from Dako and used at a dilution of 1:10,000.

### Intracellular YB-1 localisation imaging

MSTO-211H cells (2 × 10^5^) were seeded into 6-well plates and transiently transfected with 2.5 µg YB-1^EBFP2^ plasmid [11] using Lipofectamine 3000 (Invitrogen) according to the manufacturer’s instructions. On the next day, cells were stained with cell tracker green (1:5000) and 2 × 10^4^ cells were transferred into Ibidi 8-well chamber slides. After 48 h, cells were treated with 15 µM BI-D1870 or solvent and nuclei were labelled using NucRed 647 (Thermo Fisher). Images were taken 4 h later on a confocal microscope (4 × 4 stacks, 40x lens) and quantified using the Definiens Tissue Studio^TM^ Software (Definiens AG, Munich, Germany). Briefly, cell shapes were defined using green cell tracker (Thermo Fisher) and NucRed 647. Then, intensity of the YB-1 signal was attributed to each compartment (cytoplasm or nucleus) and the nuclear/cytoplasmic ratio was calculated. Only cells with YB-1 staining clearly visible on the picture were analysed. To eliminate errors from dividing or dead cells, the upper cut-off for the nuc/cyt ratio was set to 1.2.

### Drug treatment and irradiation

Cisplatin was obtained from Sigma (St. Louis, MO, USA) and dissolved in DMF. BI-D1870 was purchased from Santa Cruz (Dallas, TX, USA), LJH685 and ipatasertib were both from MedChemExpress (Sollentuna, Sweden) and dissolved in DMSO. Cell lines were irradiated with single doses of 2 Gy using a Gulmay D3300 X-ray device (Gulmay Ltd., Chertsey, UK) operated at 150 kV at a dose rate of ~1.9 Gy/min with 2.4 mm Al filter.

### Growth inhibition assays

Cells lines (2 × 10^3^ per well) were seeded into 96-well plates and cisplatin or ipatasertib and/or BI-D1870 were added as indicated on the next day. In the irradiation setting, drugs were added at the time of seeding and cells were irradiated on the next day. After 96 h (72 h drug treatment) the experiment was stopped and the proliferation of cells was analysed using a SYBR green-based assay on a Varioskan LUX Multimode Microplate Reader (Thermo Fisher Scientific) as described [19]. Combination assays were evaluated based on the HSA mathematical model of the Combenefit software [20].

### Colony formation assay

PM cells (2 × 10^3^) were seeded into 6-well plates and on the next day treated with BI-D1870 or DMSO as indicated. After 7–14 days, cells were fixed using 3:1 methanol/acetic acid and stained with crystal violet [16]. Representative images were taken on a Nikon Eclipse Ti300 microscope and cell shape descriptors (cell area and aspect ratio) were manually assessed using ImageJ as described [21]. For quantification of cell growth, cells were destained with 2% SDS and absorption measured at 562 nm on a Varioskan LUX Multimode microplate reader (Thermo Fisher Scientific).

### Cell morphology analysis

MSTO-211H cells (5 × 10^3^) were seeded into 12-well plates and on the next day treated with BI-D1870 or DMSO as indicated. After 96 h, representative bright field images were taken at ×20 magnification on an Olympus IXplore SpinSR spinning disk confocal microscope and analysed for cell size, aspect ratio, nucleus size and number of nuclei per cell using the Olympus CellSens Dimensions software. The deep learning neuronal network was previously trained on a comprehensive training dataset with manual annotation of distinct cellular features.

### Videomicroscopy, cell migration and cell fate maps

For live cell imaging, 3 × 10^3^ cells were seeded in each well of a 48-well plate. The following day, BI-D1870, LJH685 or DMSO were added at the indicated concentrations to the respective wells and live-cell imaging was started using a Nikon Visitron Live Cell System (Visitron Systems) with ×10 magnification at 10-min intervals for 96 h.

For the analysis of cell migration and average speed, at least 30 individual cells were manually tracked using ImageJ. Origin plots were created using the relative coordinates and the DiPer migration tool for Microsoft Excel [22]. For cell fate analysis, individual cell fates were manually mapped as previously described [8]. Specifically, the time a cell spent in nuclear division, and cytokinesis was assessed. Time points of other events, such as cell death, cell fission (spontaneous division of a multinucleated cell) or fusion, and abnormal cell division (division into more than two cells) were also recorded.

### Statistical analysis

Unless stated otherwise, data were analysed using GraphPad Prism 8. Differences were evaluated by Student’s *t* test or ANOVA for comparisons of two or multiple groups, respectively. Correlation analysis was performed using the Anderson–Darling test for normal distribution, and Spearman *r* was calculated accordingly. Results were considered statistically significant at *p* < 0.05. Drug interactions were evaluated using the Combenefit software, which calculates synergism scores based on the mathematical HSA model [20]. Predicted values (PV) for additive effects represent the arithmetic products of the percentage viability of each treatment alone.

## Results

### YB-1 is present in its serine 102 phosphorylated form in mesothelioma tissue and cell compartments

**Fig. 1:**
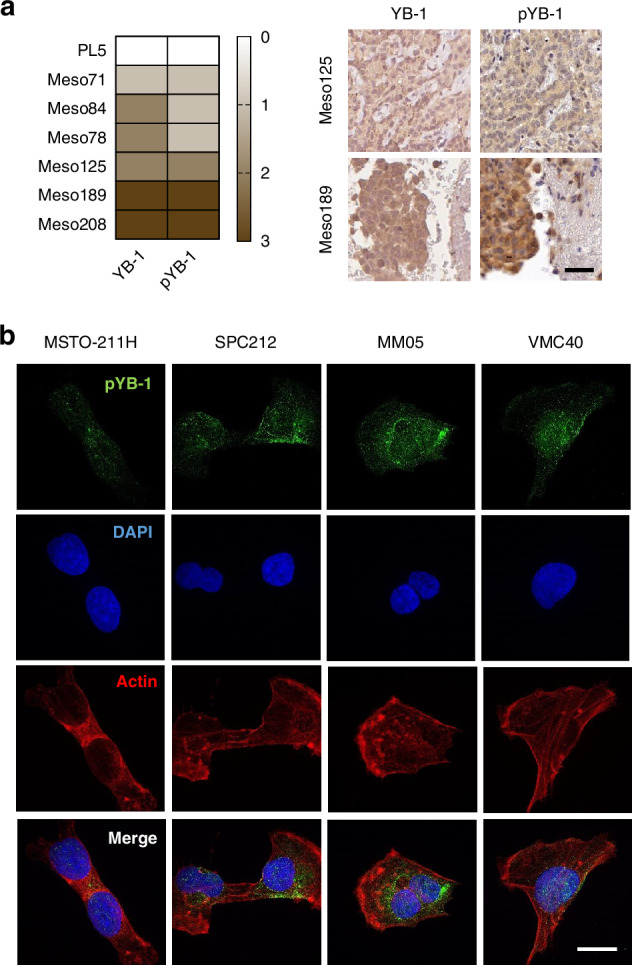
YB-1 is present in its serine 102 phosphorylated form in mesothelioma tissue and cell compartments. **a** YB-1 and phospho-YB1 (pYB-1) stainings in PM and normal pleura (PL5) tissues, scored according to staining intensities: 0 = negative, 1 = weak, 2 = medium, 3 = strong. Representative images of PM tissue stained with YB-1 and phospho-YB-1 for the scores 2 and 3. Scale bar = 50 µm. **b** Representative pictures of PM cell lines. Phospho-YB-1 (pYB-1) is shown in green. Nuclei are stained with DAPI (blue) and actin is shown in red. Scale bar = 15 µm.

Our previous studies clearly identify YB-1 as an attractive target for PM therapy [7–9], but the presence and relevance of YB-1 in its S102 phosphorylated form has not been determined. Hence, we initially analysed the expression of YB-1 and pYB-1 in PM tissue samples. IHC demonstrated the presence of pYB-1 in all specimens analysed and, moreover, YB-1 corresponded with pYB-1 positivity, indicating that a considerable part of the expressed YB-1 was indeed phosphorylated at the S102 position (Fig. 1a and Supplementary Fig. S1). Of note, both, YB-1 and pYB-1 were found in the cytoplasm and in the nucleus whereas normal pleura (PL) was negative. Next, we analysed by immunofluorescence whether pYB-1 is also detectable in PM cell lines (MSTO-211H, SPC212, MM05, VMC40) and found pYB-1 consistently present in both the cytoplasm and the nucleus of all cell lines tested (Fig. 1b).

### BI-D1870 inhibits YB-1 S102 phosphorylation and reduces its nuclear localisation

**Fig. 2:**
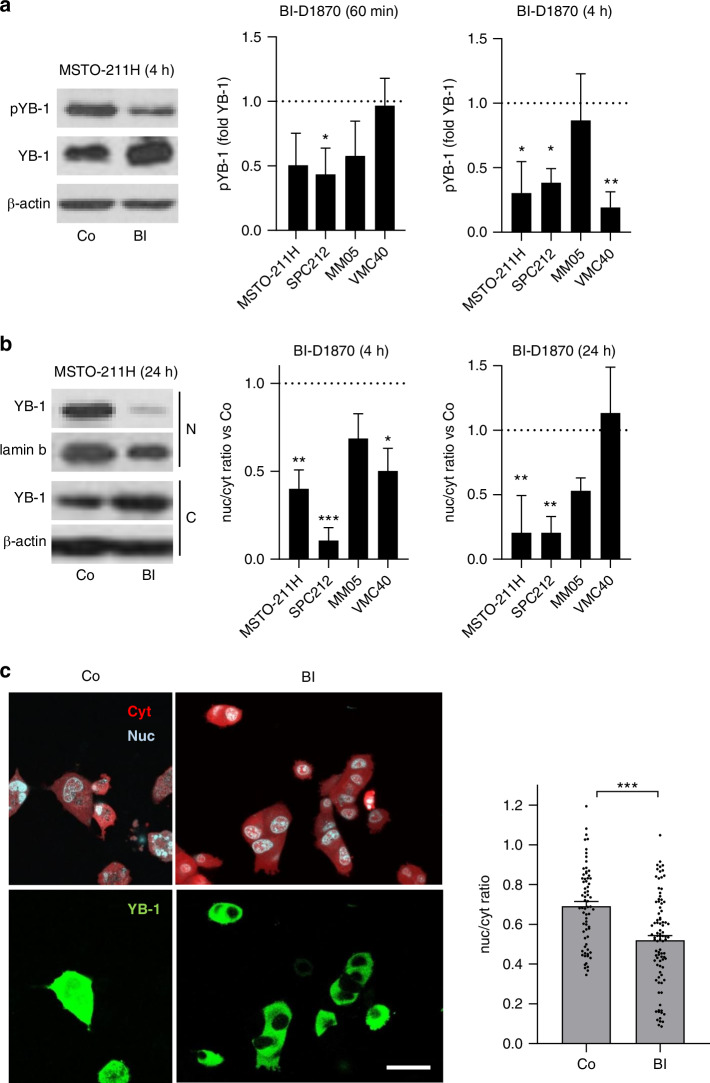
BI-D1870 inhibits YB-1 S102 phosphorylation and reduces its nuclear localisation. Representative Western blot pictures and densitometric quantification of pYB-1 in **a** total protein extracts and **b** YB-1 in nuclear and cytoplasmic fractions after the indicated times of treatment with 15 µM BI-D1870 (BI) or DMSO (Co). Beta-actin was used as loading control for total and cytoplasmic proteins, lamin b for nuclear fractions. Data are shown as mean + SEM of 3–5 replicates. *p < 0.05, **p < 0.005, ***p < 0.001. **c** Quantification of intracellular localisation and representative pictures of MSTO-211H cells transiently transfected with YB-1^EBFP2^ (green) after 4 h treatment with 15 µM BI-D1870. The nucleus and cytoplasm were counterstained with Nuc-Red (blue) and a cell tracker (red), respectively. Scale bar = 20 µm. Quantification of the nuc/cyt ration was performed automatically using the Definiens software. The data are shown as single cells and mean + SEM. ***p < 0.001.

It was previously demonstrated that YB-1 S102 phosphorylation is mediated by ribosomal S6 kinase (RSK) [23]. Therefore, we tested BI-D1870, a small molecule RSK inhibitor. Indeed, YB-1 phosphorylation was strongly reduced in PM cells after 60 min and 4 h treatment with BI-D1870 (Fig. 2a and Supplementary Fig. S2). Phosphorylation of this site was described to be essential for YB-1 nuclear localisation and the associated oncogenic properties that arise from its transcriptional activity [10, 11]. To test the effect of BI-D1870 on intracellular localisation of YB-1 in PM cells, we performed immunoblots of nuclear and cytoplasmic cell fractions. Our results show that treatment with BI-D1870 reduced YB-1 levels in the nucleus, in line with the presumptive role of S102 phosphorylation in nuclear translocation (Fig. 2b and Supplementary Fig. S3). In an alternative approach, we transiently transfected MSTO-211H cells with a blue fluorescent protein (BFP)-tagged YB-1 expression construct and observed its localisation using live cell confocal microscopy and automated image quantification establishing nuc/cyt ratios of >100 individual cells. These data confirmed that 4 h after treatment with BI-D1807, the intracellular distribution of YB-1 was significantly shifted towards the cytoplasm (Fig. 2c).

### Growth inhibition by BI-D1870 correlates with YB-1 expression and sensitivity to knockdown

**Fig. 3:**
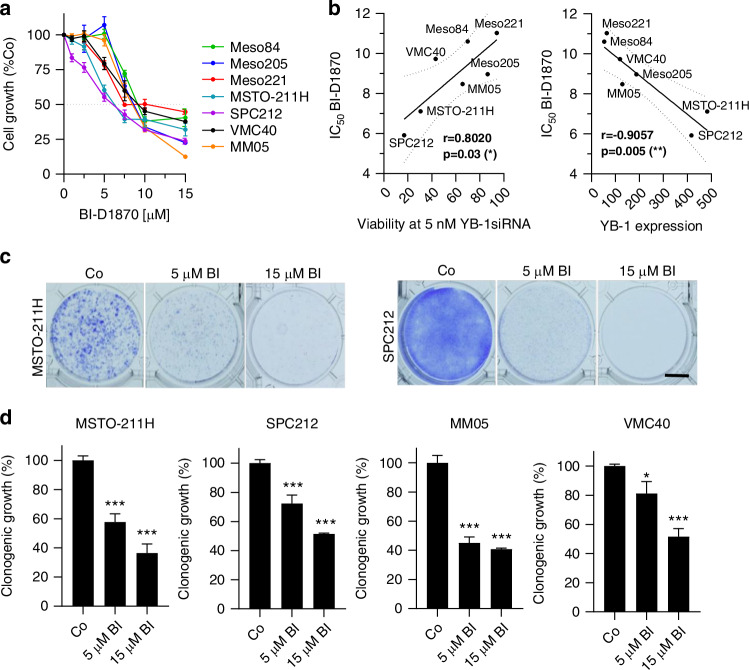
BI-D1870 reduces PM cell viability. **a** Viability of PM cells treated with BI-D1870 at the indicated concentrations for 72 h, determined by a SYBR green-based growth assay. **b** IC_50_ values of BI-D1870 after 72 h, calculated from dose-response curves in correlation with cell viability 96 h after transfection with 5 nM of YB-1-specific siRNA compared to control siRNA (left), and YB-1 expression levels normalised to the immortalised mesothelial cell line Met-5A assessed by immunoblot. Each dot represents one cell line and is the mean of at least 3 biological replicates. Pearson correlation, *p < 0.05, **p < 0.005. **c** Representative images and **d** quantification of colony formation assays treated with DMSO (Co) or 5 µM and 15 µM BI-D1870 (BI) stained with crystal violet after 7–14 days. The data are shown as mean + SEM. ***p < 0.001. Scale bar = 1 cm.

Since BI-D1870 reduced S102 phosphorylation and nuclear localisation of YB-1 in PM cells, we next tested whether BI-D1870 could replicate the growth inhibiting effect of YB-1 silencing, which has been previously published for these PM cell lines [9]. Indeed, BI-D1870 dose-dependently inhibited PM cell growth over 72 h with calculated IC_50_ values ranging from 5.9 µM to 8.9 µM in a more extended panel of PM cell lines (*N* = 7) (Fig. 3a). Sensitivity to BI-D1870 treatment strongly correlated with sensitivity to YB-1 knockdown and YB-1 expression levels that had been described in a previous publication [9], supporting pYB-1 as an important target of BI-D1870 in PM (Fig. 3b). Colony formation assays further confirmed growth inhibition by BI-D1870 in PM cells (Fig. 3c, d and Supplementary Fig. S4).

### BI-D1870 induces giant, multinucleated cells through cytokinesis failure and reduces PM cell migration

**Fig. 4:**
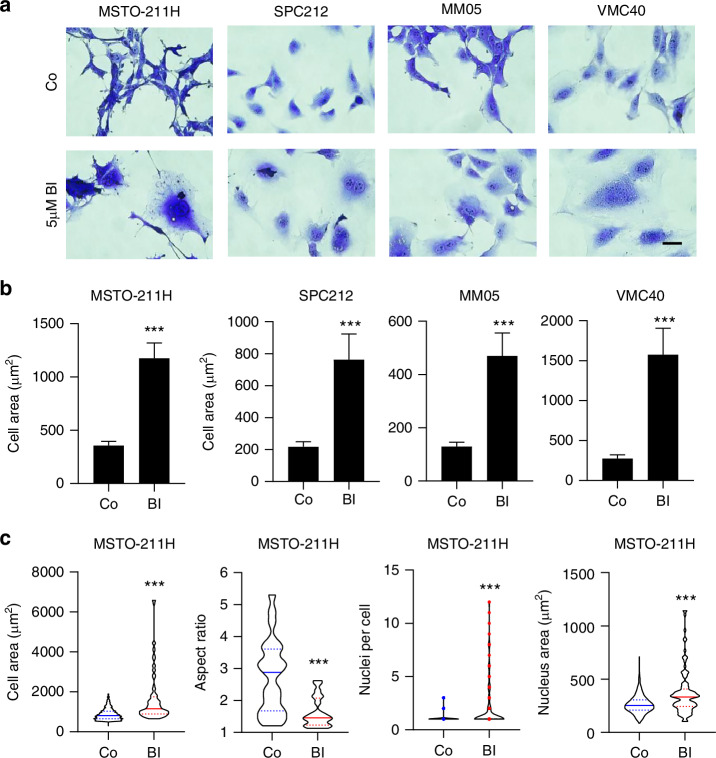
BI-D1870 (BI) changes cell morphology. **a** Representative high magnification images from the colony formation assays and **b** quantification of cell areas assessed with ImageJ. Data are shown as mean + SEM of 20–50 cells. ***p < 0.001. Scale bar = 20 µm. **c** Automated quantification of cell area, aspect ratio, nuclei per cell and nucleus area in MSTO-211H cells using the Olympus CellSens software. ***p < 0.001.

**Fig. 5:**
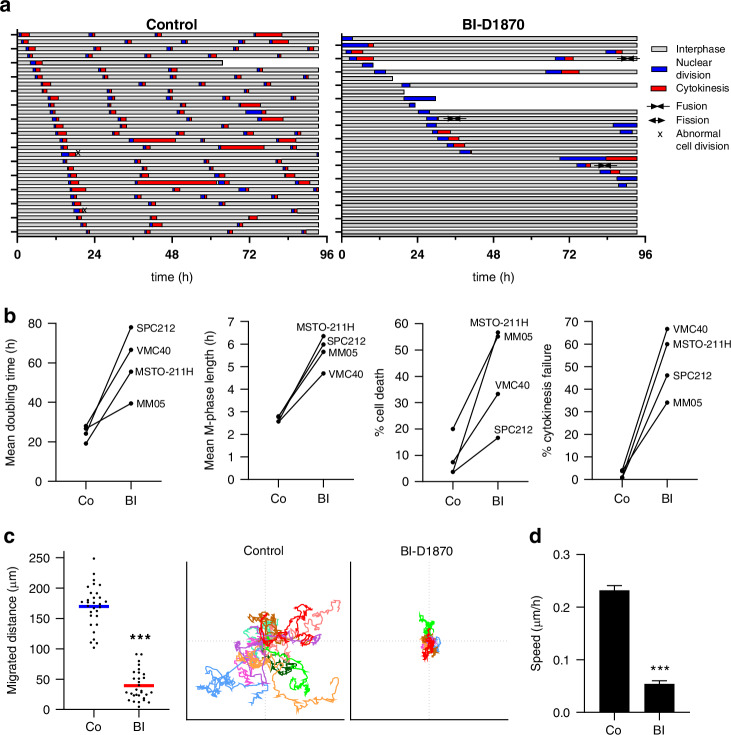
BI-D1870 induces cell cycle aberrations and reduces PM cell migration. **a** Cell fate maps of SPC212 cells, created from live-cell videos. Each bar represents one cell over 96 h treated with DMSO control or 10 µM BI-D1870, a shorter bar indicates cell death. Interphases are shown in grey, nuclear divisions in blue and cytokinesis events in red. Cellular fusions, fissions and abnormal cell divisions into more than 2 daughter cells are indicated by respective symbols. **b** Quantification of doubling time, M-phase length, % cell death and % cytokinesis failure in DMSO (Co) and BI-D1870 (BI)-treated cells, derived from the cell fate maps. Data are shown as mean. **c** Migrated distance of SPC212 cells treated with DMSO or 10 µM BI-D1870 over 72 h, derived from live-cell videomicroscopy. Each dot represents one single cell and the coloured line indicates the mean. ***p < 0.001. Origin plots of representative SPC212 cells were generated using the DiPer migration tool. **d** Average speed of SPC212 cells, assessed by DiPer. Data are shown as mean + SEM. ***p < 0.001.

Microscopic assessment of the colony formation assays revealed the formation of significantly larger and more rounded cells (Fig. 4a, b and Supplementary Fig. S5). In an alternative approach, AI-based analyses of MSTO-211H cells from microscopic images confirmed a larger cell area and reduced aspect ratio, but also showed a significantly higher number of multinucleated cells and larger cell nuclei when cells were treated with BI-D1870 for 3 days (Fig. 4c). To better characterise these effects, we generated cell fate maps from live-cell videomicroscopy data as described [21]. Tracking of at least 30 individual cells over 96 h revealed a dramatic disruption of the cell cycle in all 4 cell lines tested upon BI-D1870 treatment (Fig. 5a and Supplementary Fig. S6). Specifically, when cells were exposed to BI-D1870, we found a significant increase in doubling time and M-phase length (nuclear division plus cytokinesis) as well as cell death and cytokinesis failure events (only nuclear division), which is in line with the generation of multinucleated cells (Fig. 5b and Supplementary Fig. S7). Also, increased numbers of cell fusions or fission of multinucleated cells as well as abnormal cell divisions into more than 2 daughter cells were observed (Fig. 5a and Supplementary Fig. S6).

Since we previously demonstrated that YB-1 overexpression and knockdown in PM cells result in enhanced and diminished cell migration, respectively [24], we next tested the effect of BI-D1870 on cell migration. We found significantly reduced cell motility in response to treatment with BI-D1870, shown as cumulative migrated distance, origin plots and average speed per cell (Fig. 5c, d and Supplementary Figs. S8 and S9).

Since BI-D1870 was previously shown to inhibit Aurora B and PLK1 in addition to RSK, which could also contribute to the observed effects, we used an additional RSK inhibitor LJH685, which was demonstrated to have superior specificity for RSK compared to BI-D1870 [25]. In MSTO-211H and SPC212 cell lines, LJH685 showed comparable results to BI-D1870 with respect to reduction of YB-1 phosphorylation, inhibition of cell growth and migration (Supplementary Fig. S10). Regarding cell cycle alterations, we also found significantly increased doubling times, M-phase lengths and cell death. Cytokinesis failures, however, were slightly increased, but to a lesser extent than with BI-D1870 treatment (Supplementary Figs. S11 and S12).

### BI-D1870 positively influences the effects of cisplatin, ipatasertib and radiation treatment

**Fig. 6:**
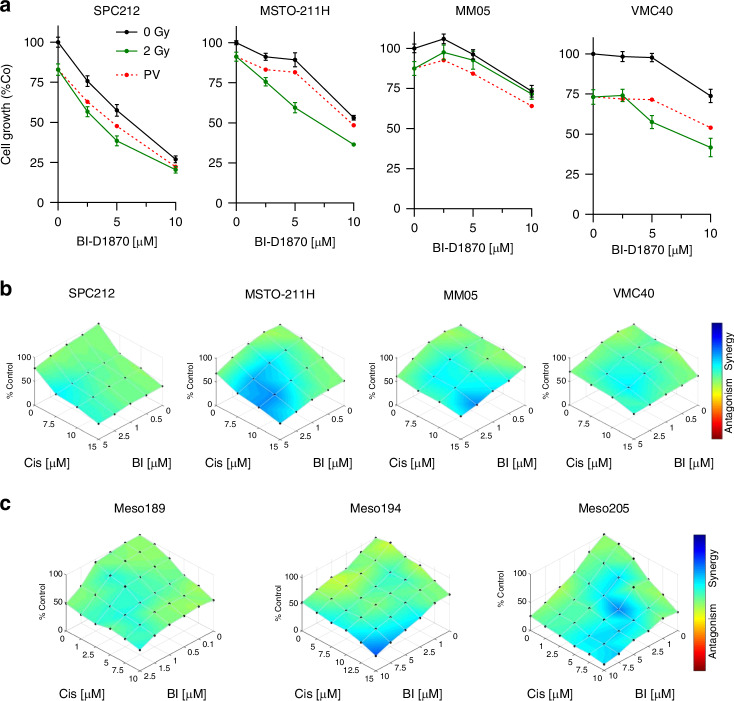
BI-D1870 positively influences the effects of cisplatin and radiation treatment. **a** Dose-response curves of cells treated with 2.5, 5 or 10 µM BI-D1870 in combination with 2 Gy or 0 Gy radiation. The red line represents the predicted value (PV) of additive effects (arithmetic product of the % viability of each treatment alone). Data are shown as mean + SEM. **b** PM cell lines and **c** primary cultures established from PM patients were treated with BI-D1870 (BI) or cisplatin (Cis) alone and in combination for 72 h and synergy maps derived from dose-response curves were created using the Combenefit software.

We and others have previously demonstrated that YB-1 plays a significant role in the response of cancer cells to cisplatin and irradiation [9, 26, 27] but no information on the role of YB-1 S102 phosphorylation on the radiation response of PM cells is available. When combining BI-D1870 with radiation at 2 Gy, we found lower than the predicted values for the combination treatment in three of the four PM cell lines tested (Fig. 6a). Since Akt has been shown to contribute to YB-1 Ser102 phosphorylation [28], and Akt signalling is frequently activated in PM [29], we tested in the MSTO-211H and SPC212 cell lines additionally the combination of BI-D1870 with the Akt inhibitor ipatasertib and found a stronger inhibition of pYB-1 by the combination treatment and additive to synergistic effects on cell viability (Supplementary Fig. S13). Finally, the combination of BI-D1870 with cisplatin, which is widely used for the treatment of PM, resulted in additive to synergistic effects with the strongest synergism found in MSTO-211H (Fig. 6b). Similar efficacy of the BI-D1870 cisplatin combination as for the PM cell lines was also observed in three primary PM cell cultures established from surgical samples (Fig. 6c).

## Discussion

The multifunctional protein YB-1 has been shown to contribute to several hallmarks of cancer and thus represents an attractive target for potential anticancer therapeutic interventions [6, 30, 31]. Since YB-1 itself does not have a kinase domain or other enzymatic functions, inhibition of YB-1 in clinical settings is not straightforward. RNA-mediated silencing of YB1 is feasible in preclinical studies and delivery of RNA-based therapeutics has also been tested in clinical trials in different malignancies including PM [32], but still poses considerable challenges. Interfering with posttranslational modifications of YB-1 that regulate its activity therefore appears as a promising alternative for blocking oncogenic functions of YB-1. In one such approach, the HDAC inhibitor entinostat was shown to enhance YB-1 acetylation, which resulted in suppression of metastasis in sarcoma [33]. Our group could recently confirm the efficacy of entinostat to block tumour growth and enhance chemosensitivity in PM and small cell lung cancer (SCLC), which in PM was clearly linked to interference with YB-1 acetylation [9, 34].

Another approach for potentially blocking oncogenic YB-1 functions is inhibiting YB-1 phosphorylation. YB-1 contains a number of phosphorylation sites including Ser102, Tyr162, Ser165, Ser167 and Tyr281 and Ser209 of which serine 102 is the most studied [6, 35]. Two kinases, RSK and Akt, have been shown to phosphorylate YB-1 at S102 [28, 36]. While many studies have demonstrated overexpression of YB-1 in different cancer types, only a fraction of those have looked at the presence of the S102 phosphorylated form and ours is the first to demonstrate S102 phosphorylated YB-1 in cell lines and histological sections of PM, which still lacks druggable targets for therapeutic interventions. Due to the activation of RSK as downstream target of the mitogen-activated protein kinase (MAPK) pathway, pYB-1 levels have been linked to Ras mutation status [37, 38]. Mutations in Ras or other oncogenic drivers are rare in PM, which is mostly characterised by loss in tumour suppressors such as BAP1, p16 and p53 [39], but several groups including ours have previously demonstrated strong MAPK activation via fibroblast growth factor receptor (FGFR) signalling in PM [16, 40].

While a correlation of YB-1 gene expression with reduced survival time of patients has been found in the TCGA dataset of PM patients [6], further effort will be required to explore potential correlations of YB-1 and pYB-1 staining patterns with patient survival or clinicopathologic characteristics in a larger cohort of PM patients. Likewise, since treatment-induced upregulation of YB-1 and pYB-1 has been found for instance in breast cancer [38, 41–43], it will be interesting to see whether prior therapy has an impact on pYB-1 and YB-1 levels in PM patient tissue. In our limited cohort, three patients (Meso84, Meso189 and Meso208) had not received any previous treatment, whereas the other three had received chemotherapy (Meso71, Meso78, Meso125), but a much larger cohort will be required to address this question.

Functionally, the blockade of YB-1 S102 phosphorylation was shown to impair stemness and survival of triple-negative breast cancer cells [10]. Our results confirm previous data linking reduced YB-1 S102 phosphorylation to reduced nuclear accumulation [12, 44]. Overall however, the regulation of YB-1 localisation in the cell is quite complex, since the importance of other phosphorylation sites, the presence of RNA in the nucleus and the cell cycle phase have all been demonstrated to play a role in the process [35, 44, 45]. Moreover, the importance of S102 phosphorylation of YB-1 for its oncogenic activity is controversial and may be cancer type specific as a study in melanoma has concluded that the unphosphorylated cytoplasmic form of YB-1 is responsible for increased metastatic potential [14].

In our current study, we have focused on the ability of the RSK inhibitor BI-D1870 to block malignant growth and enhance therapeutic responses in PM. Indeed, our data demonstrate that BI-D1870 efficiently blocked YB-1 phosphorylation at S102 and concomitantly decreased cell viability in PM cells. It is important to note that inhibition of RSK is likely to affect, in addition to YB-1, a number of other RSK downstream targets including glycogen synthase kinase 3 (GSK3), nuclear factor kappa B (NFκB) and c-Fos [46]. This implies that inhibition of targets other than YB-1 may have contributed to the observed effects of BI-D1870 in our study. Importantly however, there was a significant correlation between the activity of BI-D1870 against PM cells and the efficacy of YB-1-targeting siRNA, suggesting that YB-1 is a key target of BI-D1870 in PM. The inhibitory effects of BI-D1870 on cell migration and cell division are likewise in line with previous data using YB-1-targeting siRNA [8, 11, 24] and suggest that blocking S102 phosphorylation of YB-1 is suitable for targeting these oncogenic YB-1 traits in PM. Further support for this conclusion comes from the data with LJH685, a second and more specific inhibitor of RSK [25]. While most of the effects were similar with both RSK inhibitors, the data also highlight the likely contribution of non-RSK targets of BI-D1870. Cytokinesis failure in particular was much more frequent in BI-D1870-treated cells than in LJH68-treated cells and although it was previously reported to occur after YB-1 knockdown [11], it would also be compatible with inhibition of aurora B kinase [47], a previously identified additional target of BI-D1870 [25].

Feasibility of using RSK inhibitors in clinical settings is currently being explored. In this regard, a first-in-human phase I/Ib/2 study in patients with triple-negative breast cancer using the RSK inhibitor PMD-026 is ongoing (NCT04115306). Treatment with BI-D1870 in our study showed additive to synergistic effects when combined with radiation or with cisplatin treatment. This fits well to the demonstrated role of YB-1 in drug tolerance and general stress resistance [30, 48]. Other groups have likewise reported enhanced drug sensitivity when combining BI-D1870 with cisplatin in TMEM16A- overexpressing head and neck cancer [49], with paclitaxel in ovarian cancer cells [50] or with venetoclax/azacitidine in acute myeloid leukaemia [51].

Cisplatin and radiation in general have a greater impact on rapidly dividing cells. Since BI-D1870 led to a considerable degree of delayed or stalled cell divisions, one could have anticipated some degree of antagonistic interactions in the combination treatments, yet this was not observed in our models. Molecularly, several mechanisms have been demonstrated to confer cisplatin resistance like reduced uptake, increased efflux, sequestration by GSH, increased DNA repair and reduced propensity to undergo cell death [52]. Acquired cisplatin resistance was shown to be associated with increased nuclear localisation of YB-1 in ovarian cancer cell lines [53] and YB-1 was suggested to contribute to cisplatin resistance via the regulation of MDR1 expression in oesophageal squamous cell carcinoma and neuroblastoma cells [54, 55]. Our group has recently described that co-treatment of PM cells with cisplatin and the histone and YB-1 deacetylation inhibitor entinostat can lead to increased intracellular platinum levels [9], but the phosphorylation status and intracellular localisation of YB-1 were not investigated in that study. The data of our current study are consistent with the hypothesis that decreased nuclear YB-1 resulting from reduced S102 phosphorylation diminishes cisplatin tolerance. The mechanisms linking reduced nuclear YB-1 with cisplatin sensitivity in PM will be worked out in future studies.

Our results demonstrating that BI-D1870 enhances the response to the Akt inhibitor ipatasertib in PM cells appear promising and are in line with data showing chemo- and radiosensitizing effects in colon and breast cancer cells by combined inhibition of RSK and Akt [56, 57]. While inhibition of Akt is not currently an approved treatment for PM despite evidence of a prominent role of the PI3K/Akt axis in PM [29], these data could support testing of a recently developed triple inhibitor of RSK, Akt and S6K [43, 58] in PM.

Overall, our study clearly demonstrates that YB-1 is present in its S102 phosphorylated form in PM. Treating PM cells with the RSK inhibitor BI-D1870 results in inhibition of YB-1 phosphorylation and nuclear localisation, impairment of cell growth and migration, perturbations in cell division, and enhanced response to radiation and cisplatin. Blocking YB-1 phosphorylation with RSK inhibitors thus appears to be a promising approach for further exploration of innovative treatment strategies against PM.

## Supplementary information

Supplementary Figures S1-S13

## Data Availability

The data generated in this study are available within the article and its Supplementary Data files.
