# Supplementary material for: Inhibition of YB-1 phosphorylation enhances cisplatin activity and disrupts cell division in pleural mesothelioma
Source: Br J Cancer. 2025 Sep 4;133(9):1391–400. doi: 10.1038/s41416-025-03177-0 (PMC12572334; doi:10.1038/s41416-025-03177-0)
Supplement: Supplementary file 1 — Supplementary Figures S1-S13 [file 41416_2025_3177_MOESM1_ESM.pdf]

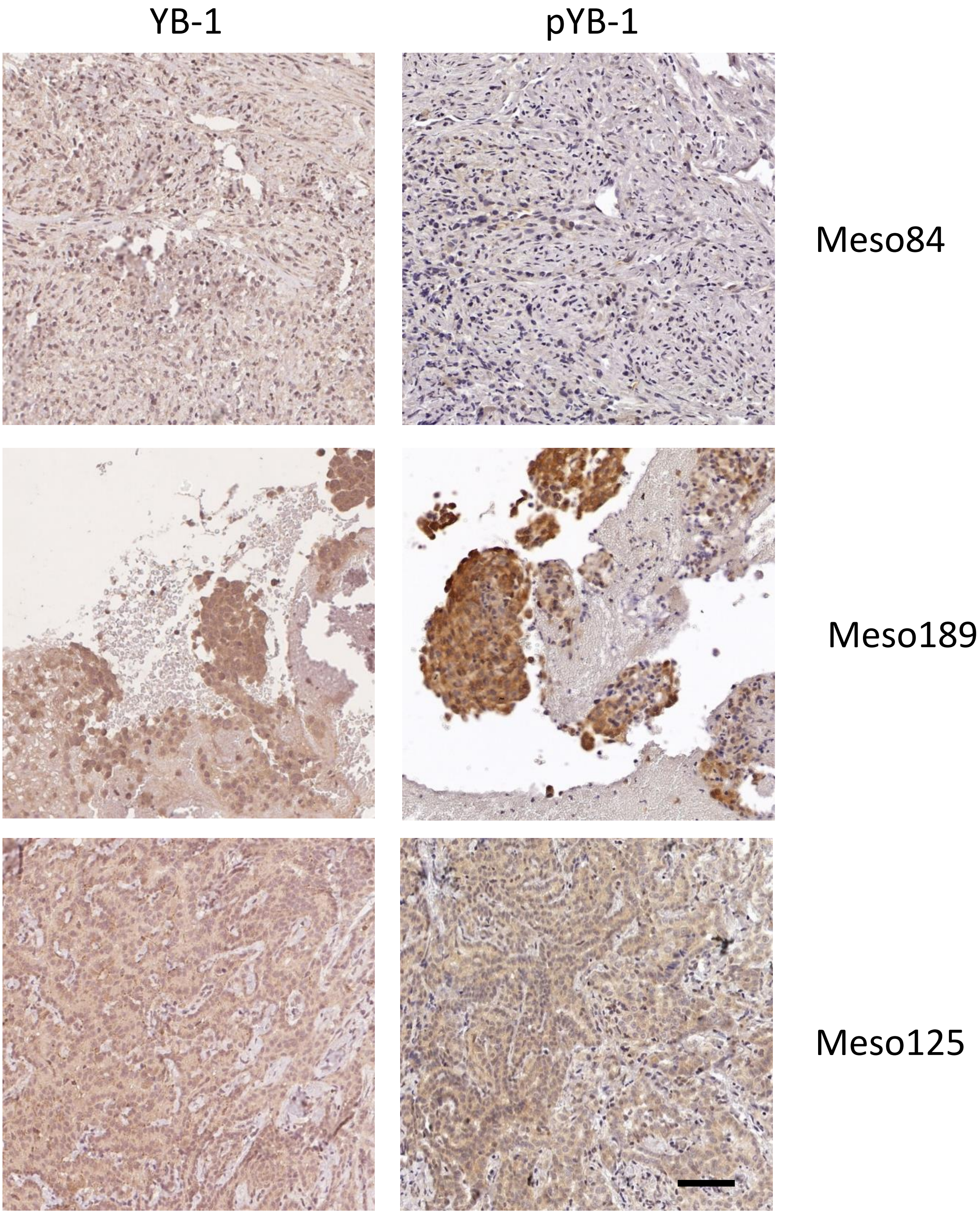

Supplementary Figure S1: YB-1 is present in its serine 102 phosphorylated form in mesothelioma tissue**.** Representative images of PM tissue stained with YB-1 and phospho-YB-1. Scale bar: 50 µm.

Schelch et al. Supplementary Figure S1

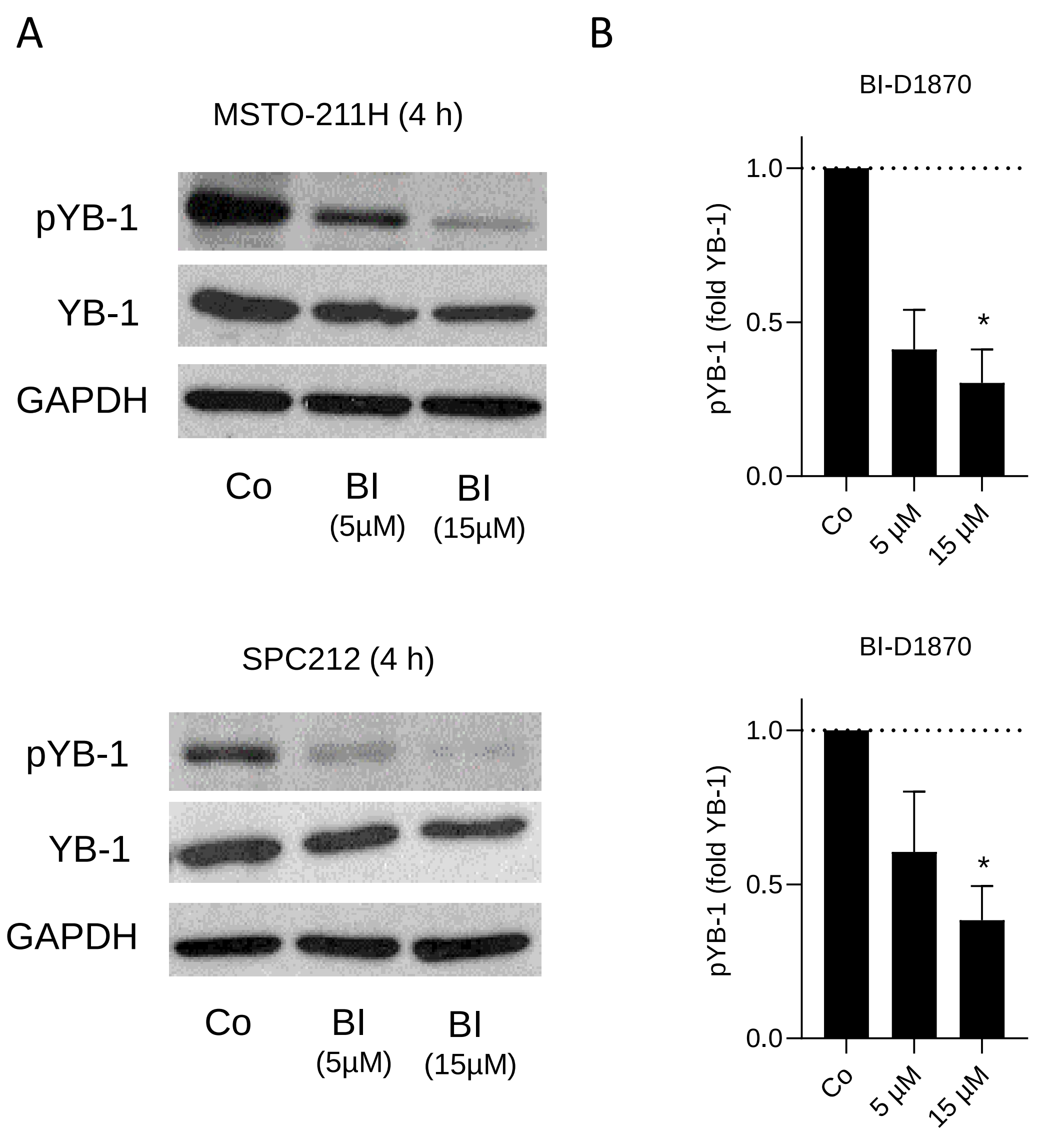

Supplementary Figure S2: BI-D1870 inhibits YB-1 S102 phosphorylation. (A) Representative western blot pictures and (B) corresponding densitometric quantification of pYB-1 after 4 h of treatment with 5 µM BI-D1870 (BI), 15 µM BI-D1870 or DMSO (Co). GAPDH was used as loading control. Data is shown as mean+SEM of at least 2 replicates.

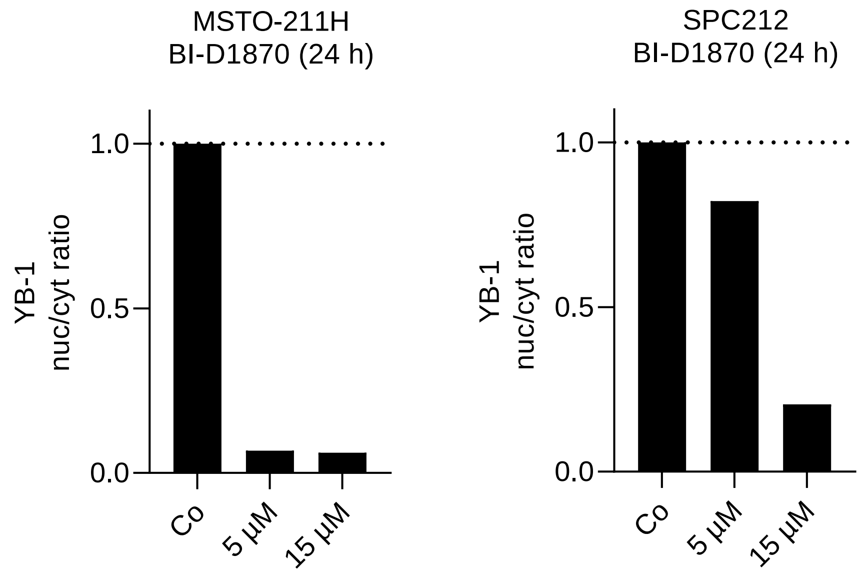

Supplementary Figure S3: BI-D1870 reduces YB-1 nuclear localization. Densitometric quantification of immunoblots detecting YB-1 nuclear to cytoplasmatic (nuc/cyt) ratio after 24 h of treatment with 5 µM BI-D1870 (BI), 15 µM BI-D1870 or DMSO (Co). GAPDH was used as loading control for total and cytoplasmic proteins, lamin b for nuclear fractions. Data is shown as mean of up to 4 replicates.

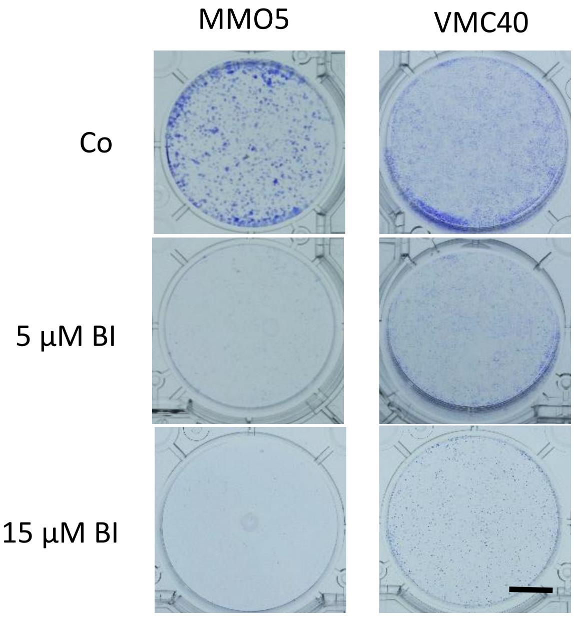

Supplementary Figure S4: BI-D1870 reduces PM cell growth. Representative images of colony formation assays treated with DMSO (Co) or 5 µM and 15 µM BI-D1870 (BI) stained with crystal violet after 7-14 days. Scale bar = 1 cm.

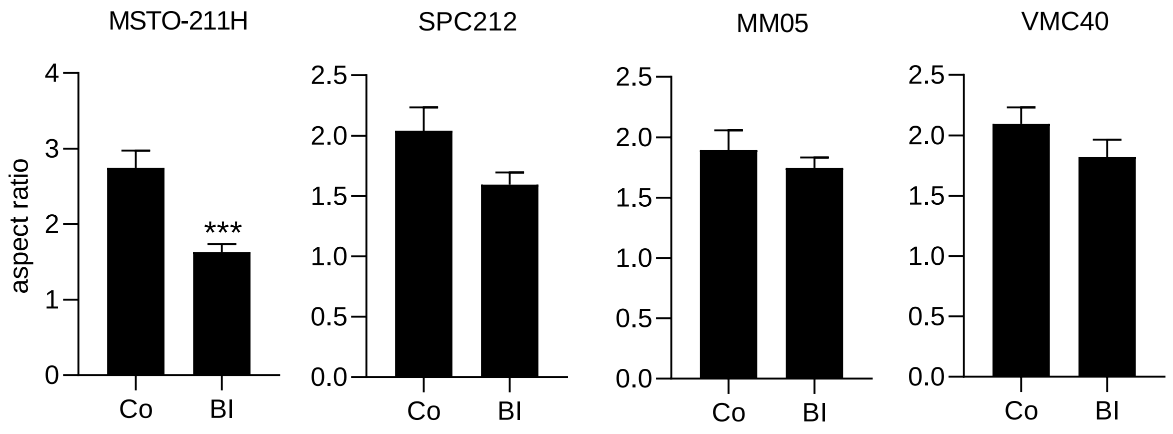

Supplementary Figure S5: BI-D1870 changes cell morphology. Aspect ratio of cells from colony formation assays treated with DMSO (Co) or 5 µM and 15 µM BI-D1870 (BI) for 7-14 days, assessed with ImageJ. Data is shown as mean +SEM of 20-50 cells. ***p<0.001.

Schelch et al. Supplementary Figure S5

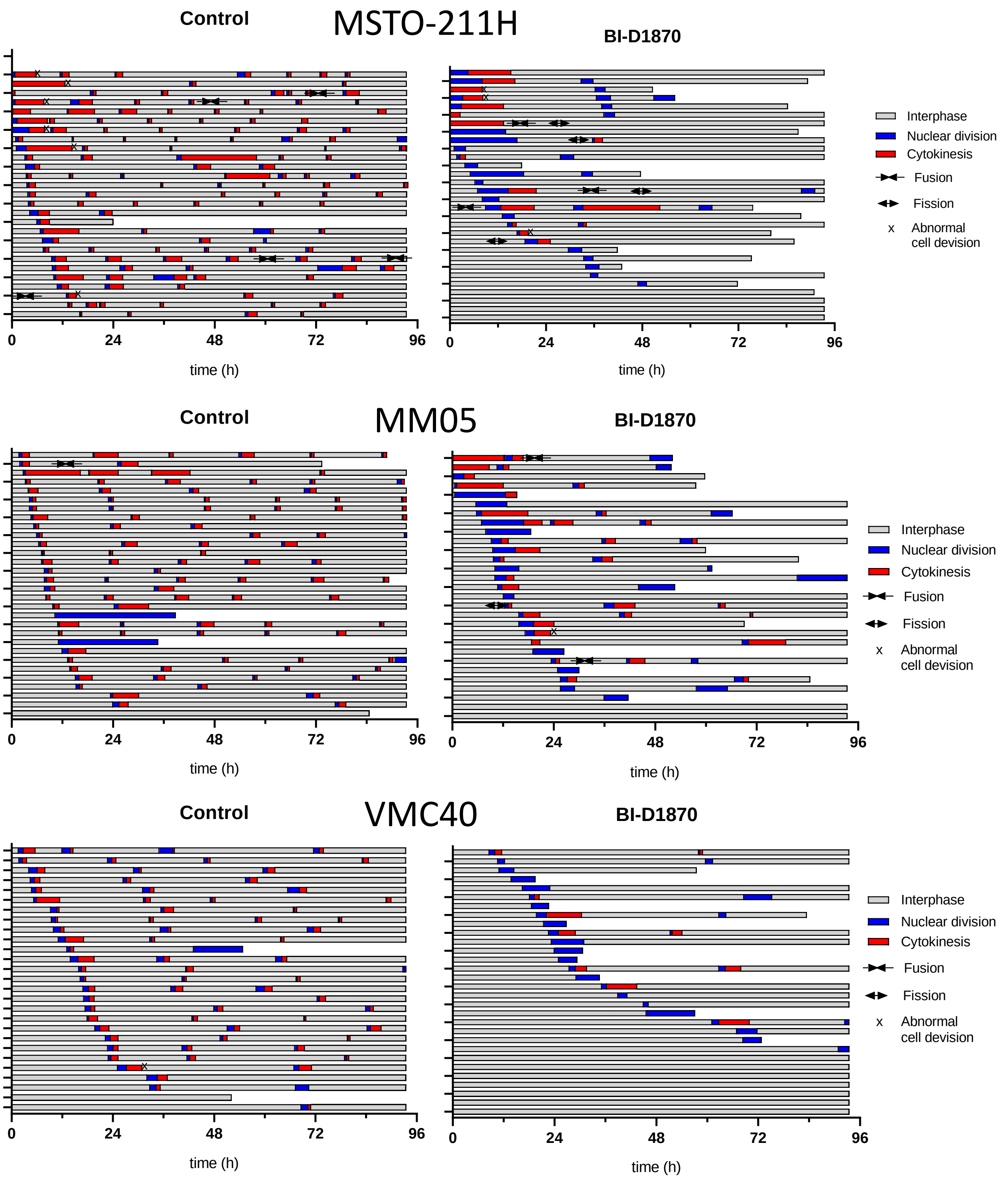

Supplementary Figure S6: BI-D1870 alters the cell cycle. Cell fate maps created from live-cell videos. Each bar represents one cell over 96 h treated with DMSO control or 10 µM BI-D1870, a shorter bar indicates cell death. Interphases are shown in grey, nuclear divisions in blue and cytokinesis events in red. Cellular fusions, fissions and abnormal cell divisions in more than 2 daughter cells are indicated by respective symbols.

Schelch et al. Supplementary Figure S6

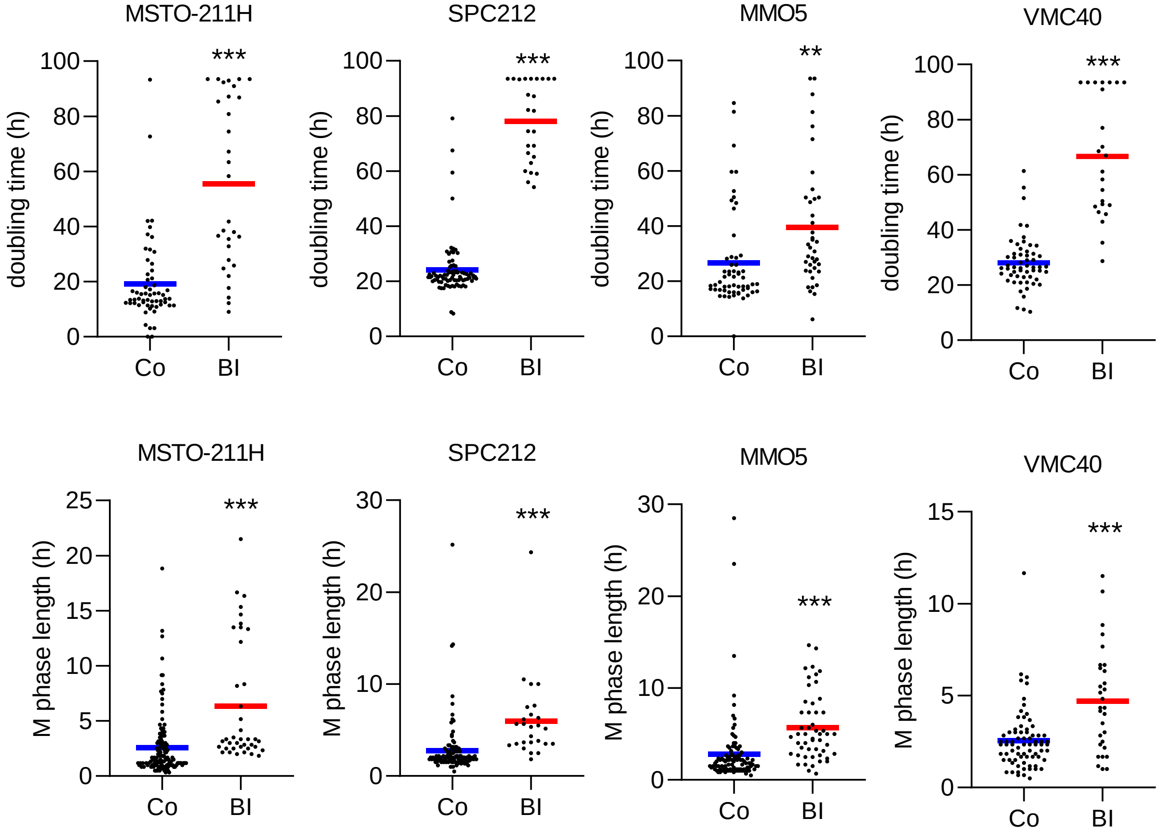

Supplementary Figure S7: BI-D1870 alters the cell cycle. Quantification of doubling time and M-phase length, derived from live-cell videos treated with DMSO control or 10 μM BI-D1870 over 96 h. Each dot represents one single cell, the line indicates the mean. **p<0.005, ***p<0.001.

Schelch et al. Supplementary Figure S7

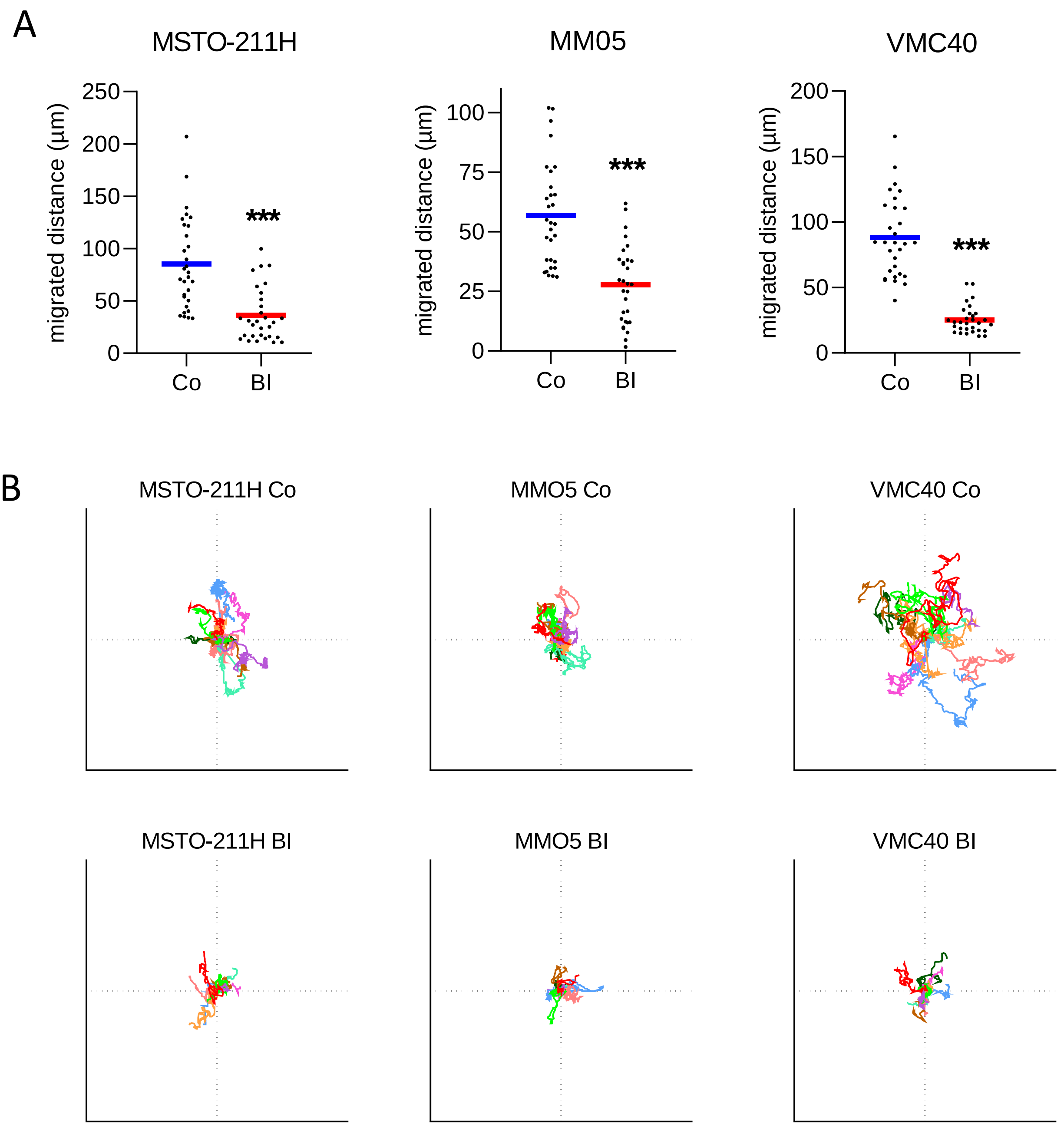

Supplementary Figure S8: BI-D1870 reduces cell migration. (A) Quantification of migrated distance with ImageJ, derived from live-cell videos treated with DMSO control or 10 µM BI-D1870 over 72 h. Each dot represents one single cell, the line indicates the mean. ***p<0.001. (B) Origin plots of representative cells were generated using the DiPer migration tool.

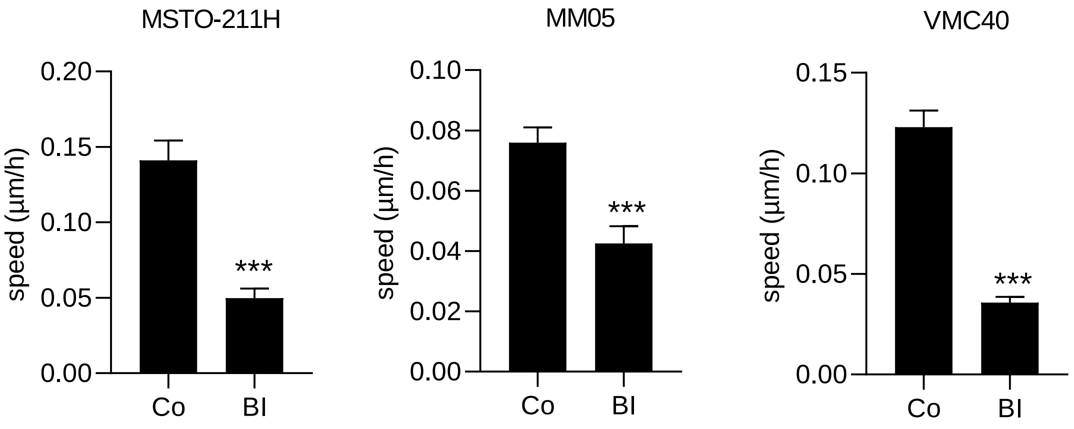

Supplementary Figure S9: BI-D1870 reduces cell migration. Quantification of speed from live-cell videos treated with DMSO control or 10 µM BI-D1870 over 72 h. Data is shown as mean +SEM. ***p<0.001.

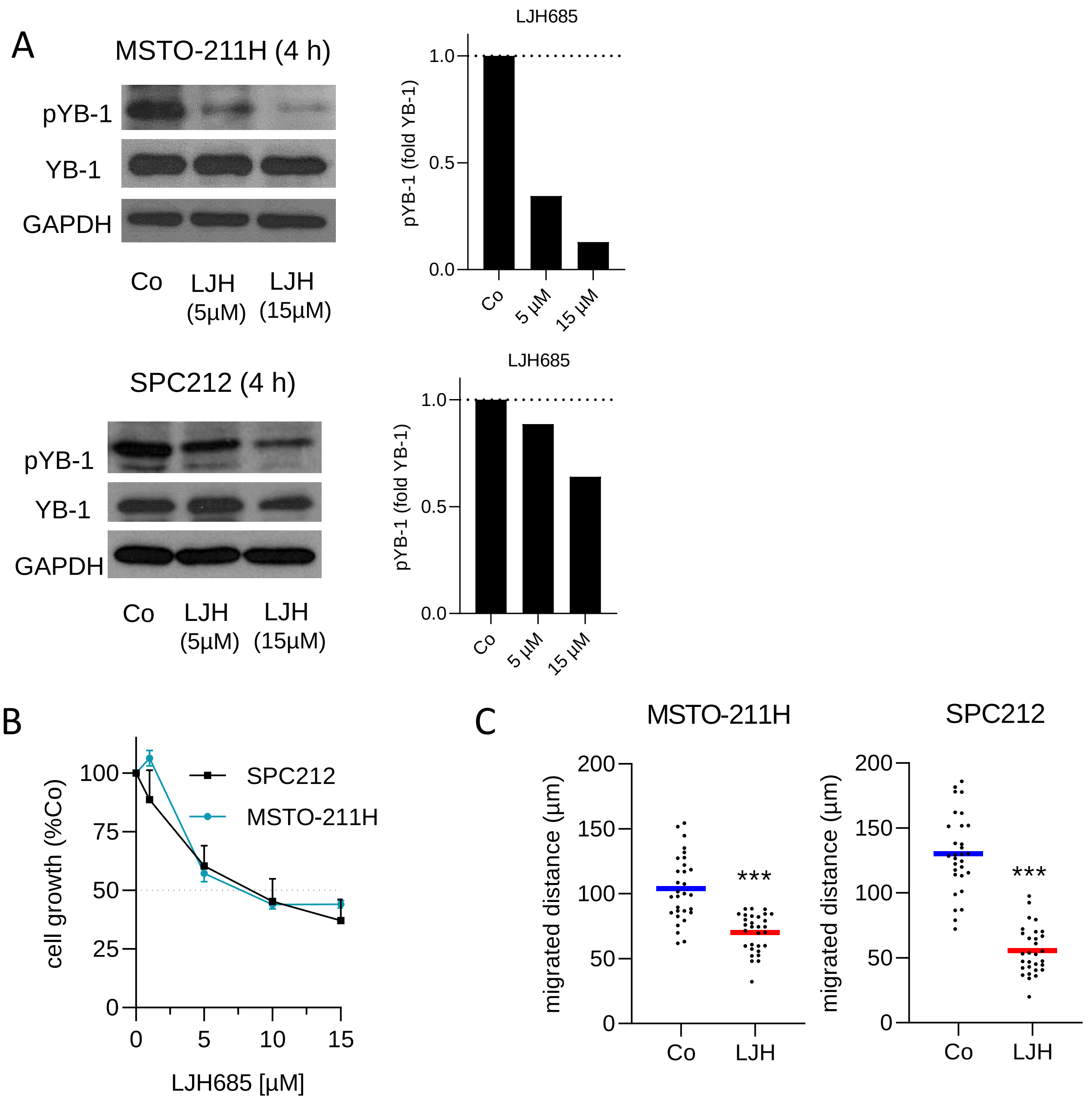

Supplementary Figure S10: LJH685 reduces YB-1 phosphorylation, cell viabiliy and migration. (A) Western blot pictures and densitometric quantification of pYB-1 after 4 h of treatment with 5 µM LJH685 (LJH), 15 µM LJH685 or DMSO (Co). GAPDH was used as loading control. (B) Viability of PM cells treated with LJH685 at the indicated concentrations for 72 h, determined by a SYBR green-based growth assay. The data is shown as mean+SEM of 4-6 biological replicates. (C) Quantification of migrated distance after 72 h treatment with DMSO (Co) or 15 µM LJH685 (LJH) with ImageJ, derived from live-cell videos. Each dot represents one single cell, the line indicates the mean. ***p<0.001.

Schelch et al. Supplementary Figure S10

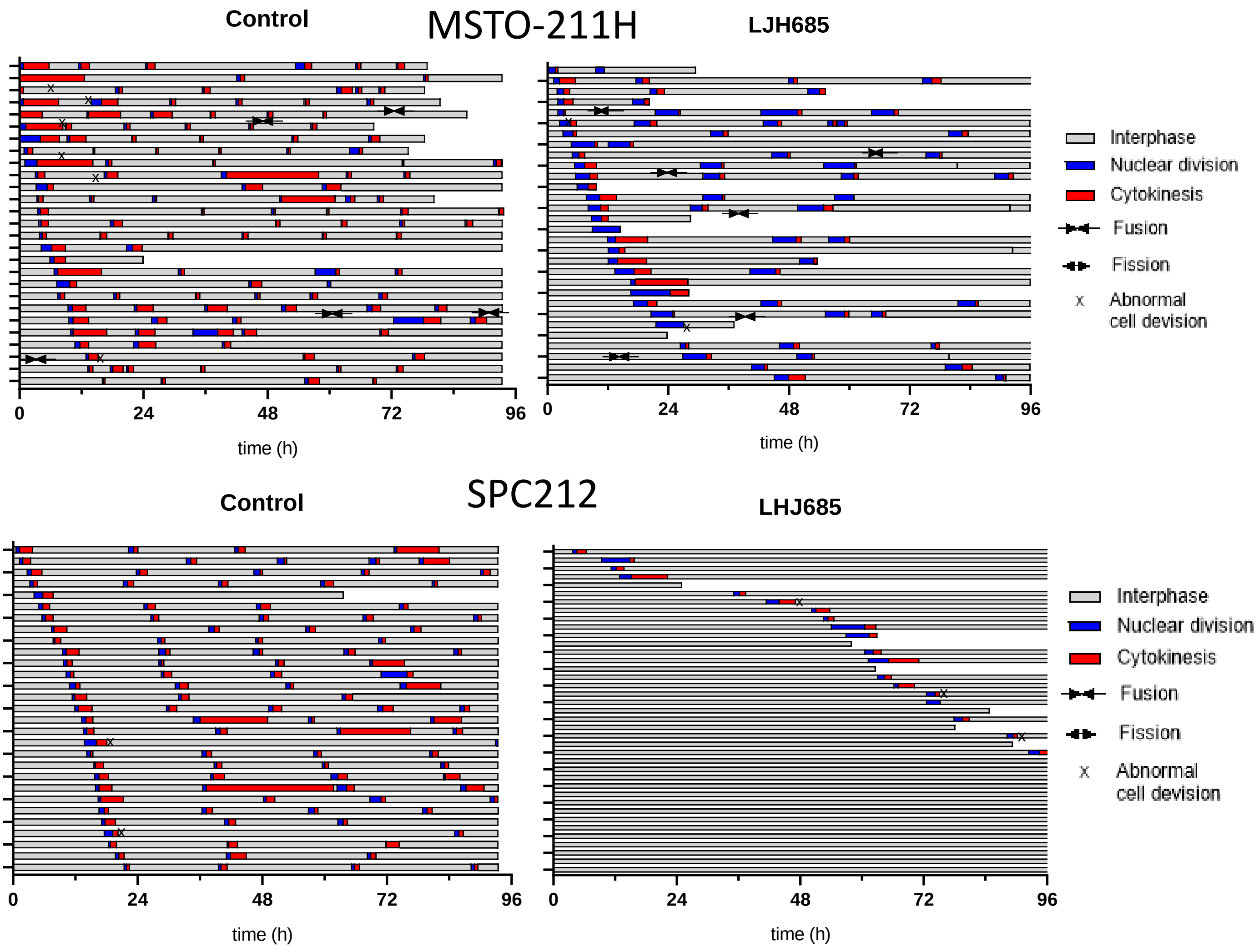

Supplementary Figure S11: LJH685 alters the cell cycle. Cell fate maps created from live-cell videos. Each bar represents one cell treated for 96 h with DMSO (Control, see Figures 5A and S6) or 15 µM LJH685, a shorter bar indicates cell death. Interphases are shown in grey, nuclear divisions in blue and cytokinesis events in red. Cellular fusions, fissions and abnormal cell divisions into more than 2 daughter cells are indicated by respective symbols.

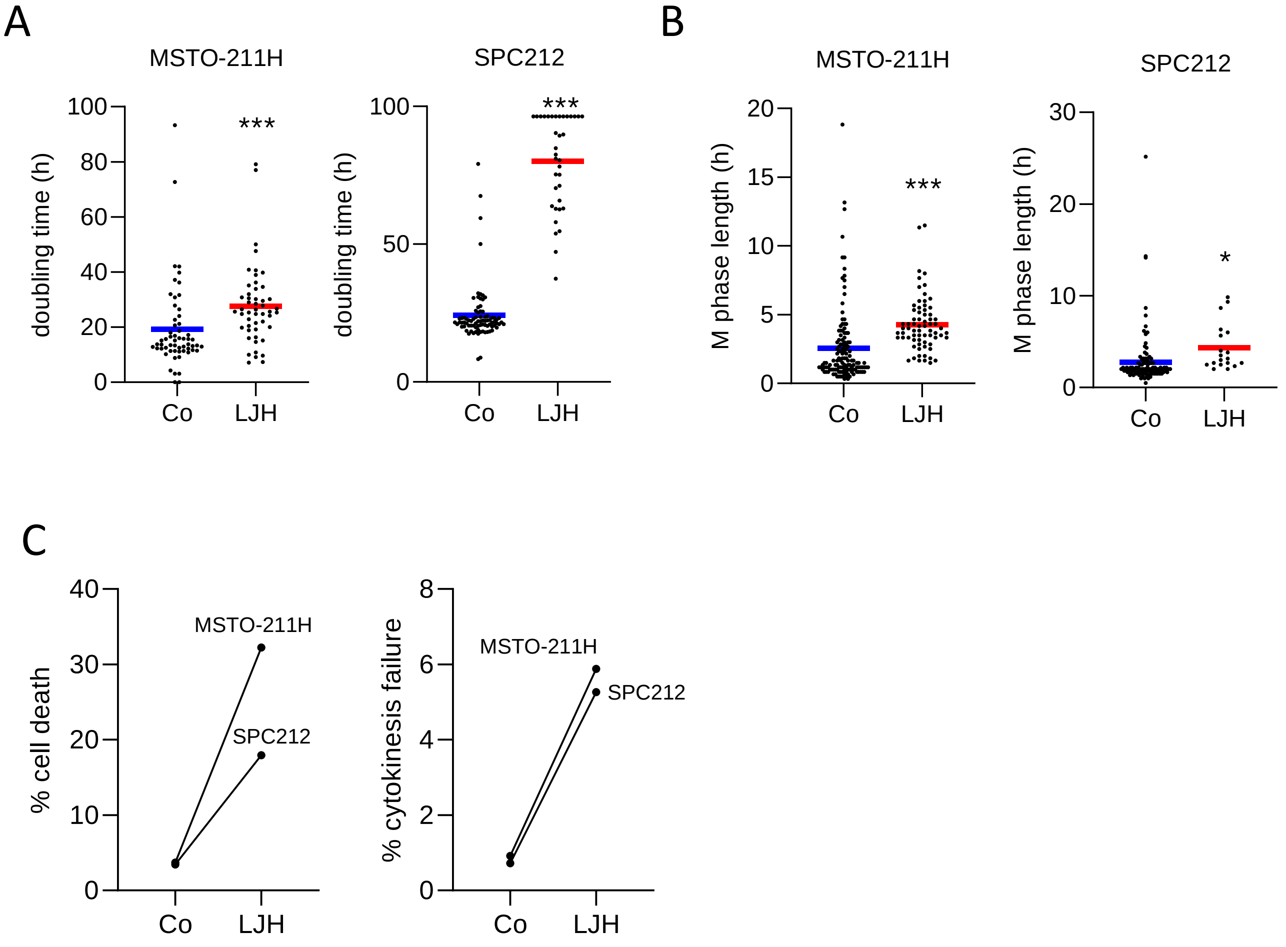

Supplementary Figure S12: LJH685 alters the cell cycle. Quantification of (A) doubling time and (B) M-phase length, derived from live-cell videos over 96 h treated with DMSO (Co, see Figures 5A and S6) or 15 µM LJH685 (LJH). Each dot represents one single cell, the line indicates the mean. *$p<0.05$, ***$p<0.001$. (C) Quantification of % cell death and % cytokinesis failure in DMSO (Co) and LJH685-treated cells, derived from the cell fate maps.

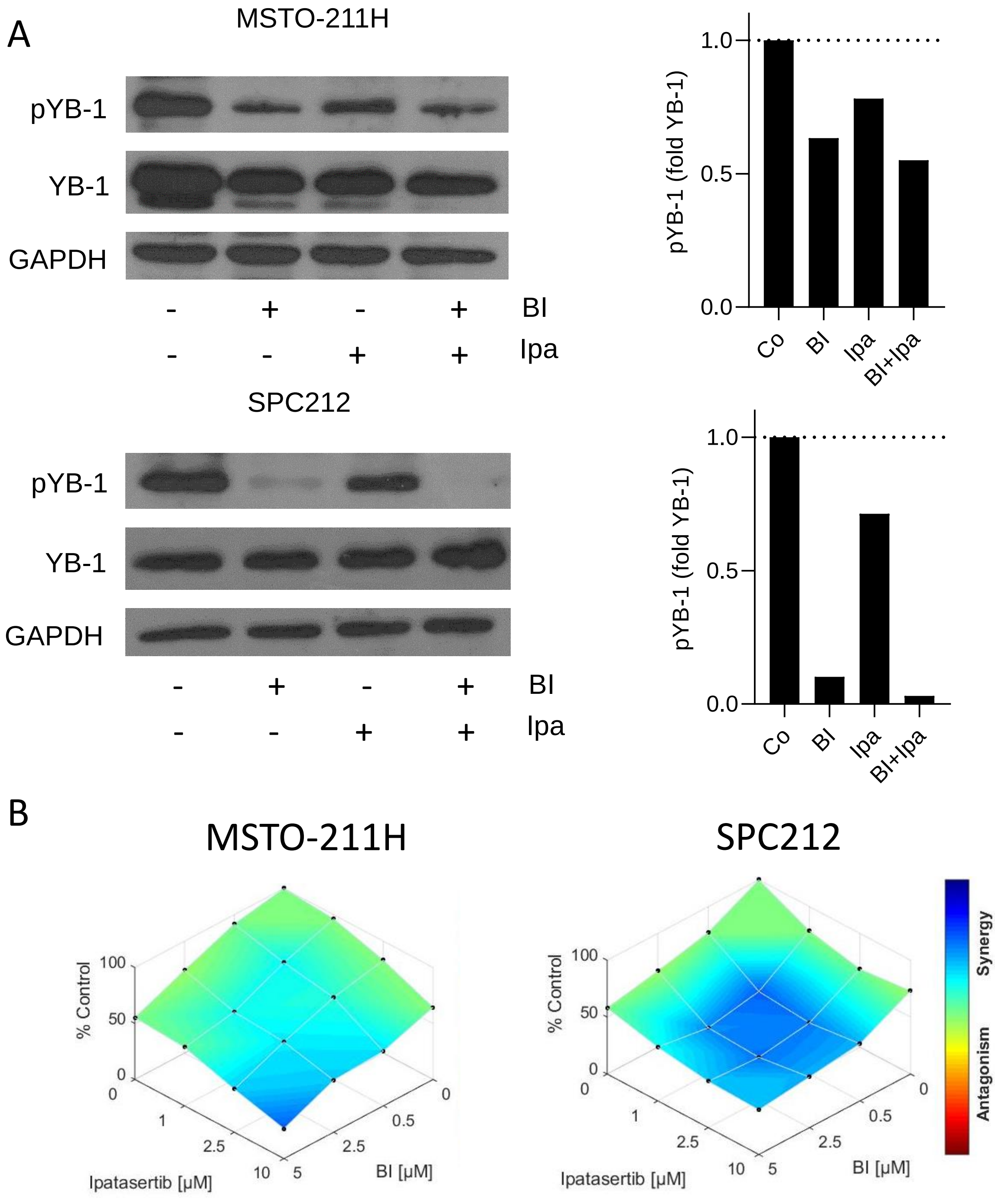

Supplementary Figure S13: Combination effects of BI-D1870 and ipatasertib. (A) Western blot images and densitometric quantification of pYB-1 after 4 h of treatment with 15 µM BI-D1870 (BI), 10 µM ipatasertib (Ipa) or DMSO (Co) as indicated. GAPDH was used as loading control. (B) Synergy maps derived from dose-response curves were created using the Combenefit software. Cells were treated with ipatasertib or BI-D1870 (BI) alone and in combination for 72 h.

Schelch et al. Supplementary Figure S13
